# Multiple functions of reversine on the biological characteristics of sheep fibroblasts

**DOI:** 10.1038/s41598-021-91468-w

**Published:** 2021-06-11

**Authors:** Yu Guo, Huan Zhu, Xiangchen Li, Caiyun Ma, Tingting Sun, Yuanyuan Wang, Chunjing Wang, Weijun Guan, Changqing Liu

**Affiliations:** 1 School of Laboratory Medicine, School of Life Sciences, Bengbu Medical College, Bengbu, 233000 China; 2 College of Animal Science and Technology, Zhejiang A&F University, Hangzhou, 311300 China; 3 Institute of Beijing Animal Science and Veterinary, Chinese Academy of Agricultural Sciences, Beijing, 100193 China; 4 Department of Neuroscience, University of Connecticut Health Center, Farmington, CT 06030 USA

**Keywords:** Cell biology, Molecular biology

## Abstract

Previous reports have demonstrated that Reversine can reverse differentiation of lineage-committed cells to mesenchymal stem cells and suppress tumors growth. However, the molecular mechanisms of antitumor activity and promoting cellular dedifferentiation for reversine have not yet been clearly elucidated. In the present study, it was demonstrated that reversine of 5 μM could induce multinucleated cells through cytokinesis failure rather than just arrested in G2 or M phase. Moreover, reversine reversed the differentiation of sheep fibroblasts into MSC-like style, and notably increased the expression of pluripotent marker genes Oct4 and MSCs-related surface antigens. The fibroblasts treated with reversine could transdifferentiate into all three germ layers cells in vitro. Most importantly, the induced β-like cells and hepatocytes had similar metabolic functions with normal cells in vivo. In addition, reversine promoted fibroblasts autophagy, ROS accumulation, mitochondrial dysfunction and cell apoptosis via the mitochondria mediated intrinsic pathway. The results of high-throughput RNA sequencing showed that most differentially expressed genes (DEGs) involved in Mismatch repair, Nucleotide excision repair and Base excision repair were significantly up-regulated in reversine treated fibroblasts, which means that high concentration of reversine will cause DNA damage and activate the DNA repair mechanism. In summary, reversine can increase the plasticity of sheep fibroblasts and suppress cell growth via the mitochondria mediated intrinsic pathway.

## Introduction

Reversine, an A3 adenosine receptor antagonist, has been shown to induce differentiated lineage-committed mouse myoblasts into multipotent mesenchymal progenitor cells^1^, promote the dedifferentiation of porcine muscle derived stem cells into female germ-like cells^2^, and transform mouse macrophages into mesenchymal progenitor-like cells^3^. Recently, we demonstrated that reversine could increase the plasticity of bovine fibroblast cells into multipotent mesenchymal progenitor cells^4^. Moreover, reversine has been shown to have an anti-cancer activity in a variety of cancer cells, such as oral squamous cell carcinoma cells^5^, acute myeloid leukemia cells^6^, thyroid cancer cells^7^, and human breast cancer cells^8^. Reversine can suppress the proliferation of multiple human cancer cells through induction of cell cycle arrest, apoptosis and autophagy^9^.

Aurora kinase family comprises serine/threonine kinases that play critical roles in cell-cycle progression, chromosome alignment and cytokinesis during mitosis, and consists of three members: Aurora A, B and C. Aberrant expression of aurora kinases has been reported in human solid and hematological cancers^10–12^. Aurora B, the catalytic subunit of chromosomal passenger complex (CPC), is involved in multiple mitotic functions, and therefore dysfunction of its kinase activity will cause segregation barrier of chromosomes, central spindle assembly impairment, and phosphorylation of histone H3^13, 14^. Moreover, as an ATP analogue, reversine exerts anti-tumor activities through the cell cycle regulators Aurora kinase, Mps1, JAK2 and SRC^15, 16^. Hence, the present study aimed reveal the potential molecular mechanisms of reversine in inducing trans-differentiation of lineage-committed sheep fibroblasts to mesenchymal stem cells and cell cycle arresting.

## Materials and methods

### Experimental animal

Small-tailed Han sheep was listed in the 138 state-level protected domestic animal breeds in China. A male sheep was provided by the Chinese Academy of Agriculture Sciences, Beijing, China. All of the laboratory animal procedures were conducted in accordance with the guidelines established by the U.S. National Institutes of Health. The protocol was approved by the Institutional Animal Care and Use Committee (IACUC) for Ethics of Bengbu Medical College (approval no. 2020–025). All sections of this report adhere to the ARRIVE Guidelines 2.0 for reporting animal research^17^.

### Effects of reversine on the proliferation and differentiation of fibroblasts

Reversine (CAS NO. 656820–32-5) was purchased from Sigma-Aldrich (Merck KGaA, Darmstadt, Germany). The Small-tailed Han sheep adult fibroblasts (SAFs) from ear marginal tissue were cultured using the tissue adherent culturing method and enzyme digestion. The cells were maintained in high glucose-DMEM (H-DMEM) medium supplemented with 10% fetal bovine serum (Gibco, Thermo Fisher Scientific, MA, USA), 1% non-essential amino acids (NEAA), 100 U/mL penicillin and 0.1 mg/mL streptomycin, and cultured at 37.5℃ in a 5% CO_2_ humidified atmosphere^4^. The fibroblasts (below passage 6) in the logarithmic phase were treated with reversine (100 nM, 500 nM, 1 μM, 5 μM and 10 μM) for 4 days. Negative control cultures were maintained in the same volume of DMSO without reversine.

### Reversine treatment and cell differentiation potential assays

The fibroblasts (2000 cells/cm^2^) were pretreated with 5 μM of reversine for 3 days, and then further induced under lineage-specific inducing conditions^18^. Osteoblasts-inducing differentiation (OID) medium: Low glucose-DMEM (L-DMEM) supplemented with 10% FBS, 50 mg/mL ascorbic acid-2-phosphate (AsA2P), 1 mM dexamethasone and 10 mM β-glycerophosphate for 14–21 days, and osteogenesis was assessed by alizarin red staining; Adipocytes-inducing differentiation (AID) medium: H-DMEM supplemented with 1.7 mM insulin, 1 mM dexamethasone, and 0.5 mM 3-isobutyl-1-methylxanthinethe (IBMX) for 7 days, and adipogenesis was assessed by Oil red staining; Neurocytes-inducing differentiation (NID) medium: DMEM/F-12 supplemented with the ITS and 0.5 μM all-trans-retinoic acid (RA) for 2 days, and then switched into serum-free medium in the absence of RA for 14 days; Hepatocytes-inducing differentiation (HID) medium: L-DMEM supplemented with 5% FBS, 20 ng/mL IGF-I, 20 ng/mL HGF and 10^–7^ M dexamethasone for 21 days. Hepatocytes differentiation was evaluated by Periodic acid-Schiff (PAS) glucogen staining. Islet-like clusters containing insulin-producing β-like cells were confirmed by dithizone (DTZ) staining (1:200, Bioss, Beijing, China). The concentrations of urea and albumin were assayed in supernatants using enzyme-linked immunosorbent assay (ELISA).

### Assays of cell skeleton and intracellular ROS

For F-actin detection, cells were incubated with phalloidin-FITC for 30 min at room temperature. For α-tubulin detection, the cells were incubated with anti-α-tubulin primary antibody (1:100, Abcam, Shanghai, China) for 1 h, and then incubated with CY3-conjugated secondary antibody (1:500, Jackson Labs Technologies, USA) for 1 h. The results of cell skeleton immunofluorescence were observed under confocal microscopy (TE-2000-E, Nikon, Tokyo, Japan). The intracellular ROS were examined using DCFH-DA and Rh123 staining, respectively^19, 20^.

### Measurement of multinucleated cells and G-band analysis

The SAFs in the logarithmic phase were treated with reversine(5 μM) for 4 days. The nuclei were stained with 1 µg/mL DAPI (Sigma-Aldrich) for 20 min at room temperature. The morphology of multi-nuclei (two or more nuclei in one cell) was confirmed under a fluorescent microscope. Chromosomes spreads were prepared, fixed and stained following standard methods^4^. After Giemsa staining, 100 well-spread metaphases were observed under an oil immersion objective.

### Immunofluorescence analysis

Cells grown on coverslips were fixed with 4% paraformaldehyde, followed by permeabilization with 0.2% Triton X-100 for 10 min, and blocked with 1% bovine serum albumin (BSA) and 10% Normal Donkey Serum(NDS) or 10% Normal Goat Serum(NGS) for 1 h at RT^4^. The following primary antibodies were then used to incubate with the cells overnight at 4 °C, the neuronal markers: anti-NFM, anti-MAP2, anti-TuJ-1 (1:200, Abcam), anti-NSE and anti-GFAP(1:200, Neuromics, MN, USA); cell surface markers: anti-CD29, CD44 (1:100, Abcam) and CD71, CD73 (1:100, Santa Cruz, CA, USA); the epigenetic markers: anti-histone acH3K9(1:200, Santa Cruz), anti-histone meH3K9(1:200, Abcam), anti-phosphor-Histone H3S10(1:1,000, Santa Cruz); the pluripotent markers: anti-Oct4, anti-Sox2 and anti-Nanog (1:200, Cell Signaling Technology, Danvers, MA, USA)^4, 18^. After that, the cells were incubated with CY3/488/543-labeled secondary antibody (Invitrogen, CA, USA) for 1 h, and the results of immunofluorescence were observed under confocal microscopy. The catalog numbers for all primary antibodies used in this research are presented in Table S1.

### Flow cytometry instrument detection

The cell cycle distributions of the DMSO and reversine-treated cells fixed with 70% ethanol and stained with PI were analyzed using flow cytometry. The effects of reversine on the expression of cell surface markers and epigenetic markers were detected following by colabelling with primary antibodies of CD29, CD44, CD71, CD73, OCT4, acH3K9, meH3K9 and p-H3S10 using flow cytometry, respectively^4^. The proportions of apoptotic cells were evaluated using an Annexin V/FITC staining kit (Beyotime, Jiangsu, China).

### Western-blot analysis and quantitative real-time PCR

For western blot analysis, equal amounts of proteins for each sample were separated by SDS-PAGE and then electroblotted onto PVDF membranes (0.45 μM, Millipore, USA), and immunoblotted overnight at 4 °C with primary antibodies^21^. The membranes were then incubated with anti-rabbit peroxidase-conjugated secondary antibody (1:5000, Jackson Labs), visualized with Clarity Western ECL Blotting Substrates (Bio-Rad Laboratories, USA)^22^. The relative quantitative PCRs (qPCR) were carried out with SYBR Premix Ex Taq kit (TaKaRa Bio, Dalian, China) on a QuantStudio6 Flex thermocycler (Applied Biosystems, Carlsbad, CA, USA)^2^, and the primers used in PCR reaction are presented in Table S2.

### Bioinformatics analysis on RNA-Seq and qRT-PCR validation

Total RNAs were extracted from four samples, DMSO control (DC), Reversine 1 days (R1), Reversine 4 days (R4) and Reversine 4 days/Rescue 15 days (D15), using the Arcturus PicoPure RNA Isolation Kit (Applied Biosystems). The integrity of total RNAs was evaluated using an Agilent 2100 Bioanalyzer (Agilent Technology, USA). Four cDNA libraries were constructed at Sangon Biotech Co., Ltd (Shanghai, China), subjected to 125 bp end sequencing using an Illumina HiSeq 2500 platform (Illumina, San Diego, CA, USA).

Raw reads were filtered to get high quality clean reads. Each sample was then mapped to a reference genome with TopHat2 (version 2.0.3.12). The expression levels were normalized by FPKM (Fragments Per Kilobase of transcript per Million mapped reads) method^23^. DESeq (an R package http://www.rproject.org/) was used to evaluate differentially expressed genes (DEGs) in four groups, of which genes with a fold change ≥ 2 and a false discovery rate < 0.05 were deemed to be the significant DEGs^24^. The Gene Ontology(GO) classifications were compared between the up-regulated and down-regulated unigenes using the WEGO method. “Path_finder” software was used to annotate the pathways related to the DEGs and compared against the KEGG database^25^. A portion of gene expressions were detected by qPCR, not merely verifying the reliability of sequencing data, but also sustaining the legitimate inferences based on the above analysis^26^.

### Statistical analysis

All data are expressed as the mean ± SD from at least three independent experiments. The Student’s t-test was used to determine statistical significance (*P* < 0.05). One-way ANOVA was performed to compare more than two groups. The software of GraphPad Prism 7.0 (San Diego, CA, USA) was used for statistical analysis and the generation of graphs.

## Results

### Effects of reversine on the biological characterization of sheep fibroblasts

**Figure 1:**
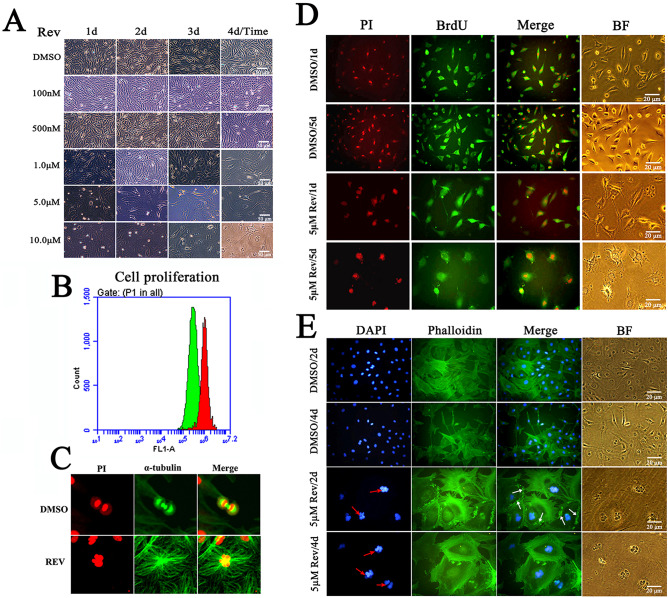
Effects of reversine on the biological characterization of sheep fibroblasts. (**A**) Effects of different concentrations of reversine on the morphology of fibroblasts for 4 days. (**B**) Cell proliferation was detected using flow cytometry. (**C**) Expression of α-tubulin (Green) was analyzed by immunofluorescence and counterstained with PI (red); (**D**) Immunodetection of BrdU incorporation for cell proliferation analysis. (**E**) Cells were stained with phalloidin (green), and counterstained with DAPI (blue). Multinucleated giant cells strongly expanded actin network and stress fibers.

There was no significant difference in size and morphology between treated cells with 0.1–1.0 μM reversine and control cells. However, after 4 days of treatment with 5 μM or 10 μM of reversine, sheep fibroblasts acquired a notably different morphology, and transformed into hypertrophic, flattening, epithelial-like, and more adhesion (Fig. 1A, Fig. S1A). Cell viability significantly decreased in cells treated with reversine in a time- and dose-dependent manner (Fig. S1B). Cell proliferation was also completely inhibited by reversine in a dose-dependent manner. The cell proliferation rate reduced by 50% after treated with 5 μM reversine for 4 days through flow cytometry detection and BrdU indirect immunofluorescence (Fig. 1B,D).

The fibroblasts treated with reversine displayed a marked alteration of actin stress fiber and tubulin organization, as detected via phalloidin and tubulin labeling staining, respectively (Fig. 1C,E). Reversine treatment resulted in many of fibroblast cells became flattened giant cells with multiple nuclei (Fig. 1E red arrows), and strongly expanded actin network. Moreover, high concentrations of F-actin were observed as aggregates or ring-like structures at the periphery of reversine treated cells (Fig. 1E). Multinucleated giant cells strongly expanded actin network and stress fibers as well as F-actin aggregation (Fig. 1E white arrows). However, the nuclei of most treated cells did not exhibit apoptotic features, such as nuclear fragmentation, chromatin condensation and margination as well (Fig. 1E).

**Figure 2:**
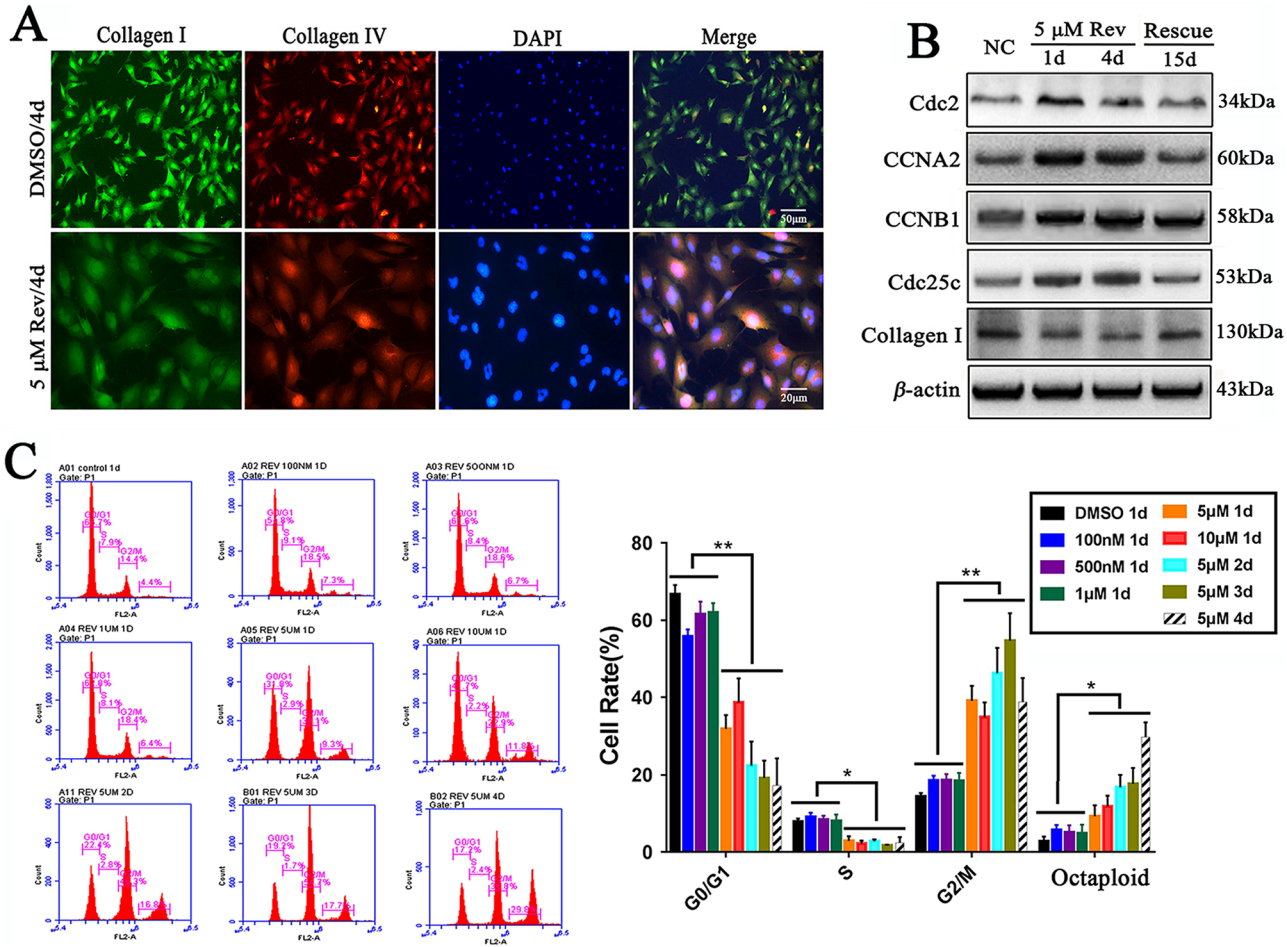
Cell cycle and ECM proteins alteration in reversine treated fibroblasts. (**A**) Reversine reduced the expression of collagen-I and collagen-IV proteins. (**B**) Reversine treatment promoted the expression of G2/M regulatory proteins, including Cdc2, Cyclin A2, Cyclin B1 and Cdc25c. (**C**) Fibroblasts treated with reversine exhibited significantly difference in the percentages of sub-G1, G0/G1, S, and G2/M phase distributions.

Immunofluorescence assay showed that the expression of collagen type I and collagen type IV was decreased after treatment with > 5 μM reversine (Fig. 2A). Consistently, western blot analysis also showed that reversine significantly suppressed collagen type I expression (Fig. 2B). In addition, 17 DEGs involved in ECM-receptor interaction pathway were significantly down-regulated in Reversine treated fibroblasts by RNA sequencing, including COL1A1, COL1A2 and COL4A1 (Fig. 9B,C). Therefore, reversine might promote the degradation of ECM and attenuate the expressions of collagen type I.

### Reversine induces multinucleation of sheep fibroblasts

**Figure 3:**
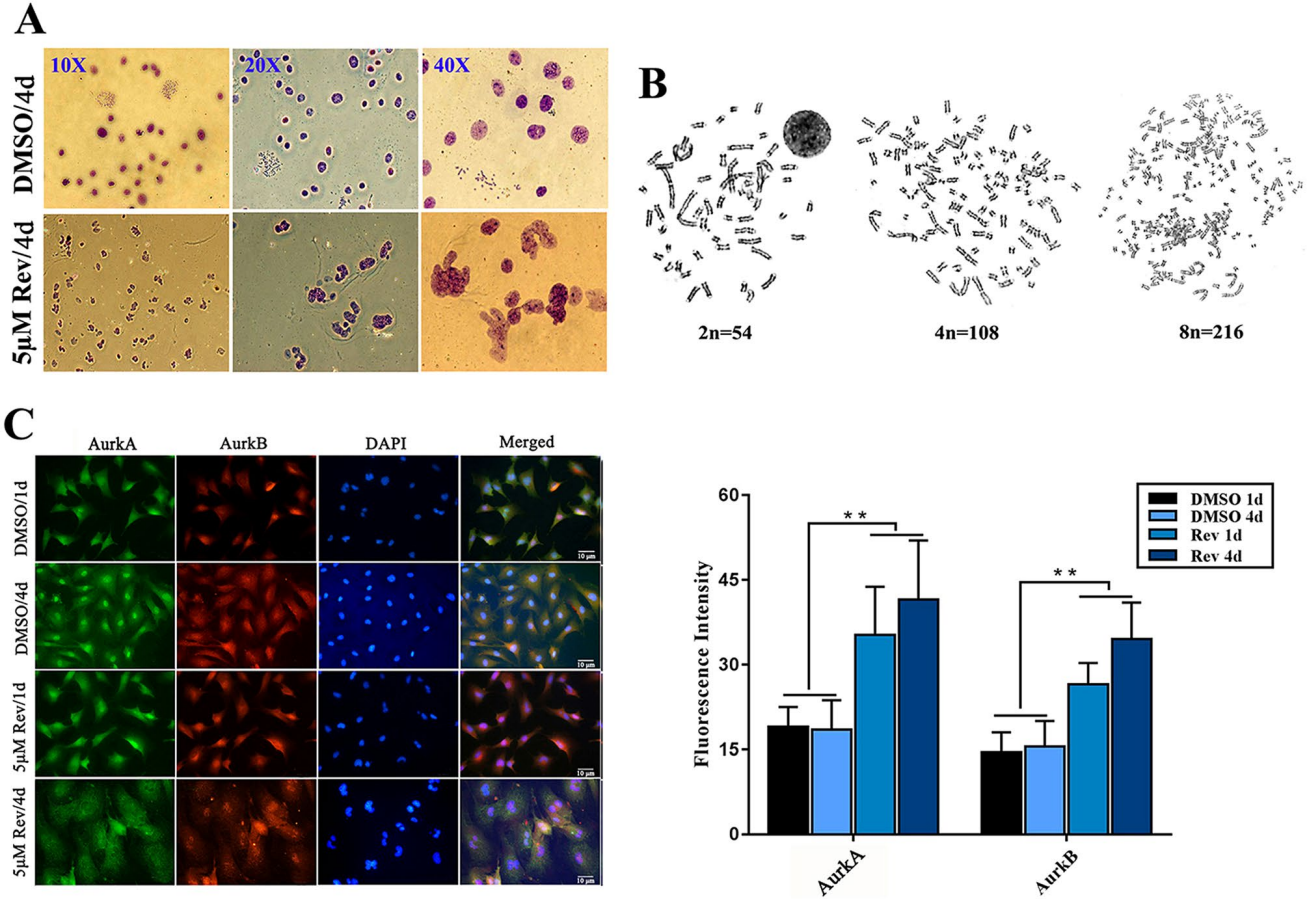
Reversine treatment significantly increases the number of cells over tetraploid. (**A**) Multinucleated cells were detected using chromosome Gimea staining. (**B**) Chromosome G-banding analysis verified the conversion of reversine-treated cells from diploid/mononuclear (2 N = 54) to tetraploid/binuclear (4 N = 108) and octaploid (8 N = 216). (**C**) The expression of Aurora A and Aurora B gradually increased with reversine treatment for 4 days by immunofluorescence.

Reversine treatment resulted in a significant decrease of cells in the G0/G1 phase which significantly decreased from 66.7% in control group to 31.0% in 5 μ M group and 41.7% in 10 μM group after 24 h of reversine treatment (*P* < *0.05*), respectively. The number of cells in the S phase also decreased (*P* < 0.01) from 7.9% to 2.9% and 2.2%, while the cells in G2/M phase increased significantly from 14.4% to 39.1% and 22.9% (*P* < 0.01) (Fig. 2C). Thus, the enrichment of tetraploid cells was promoted by reversine treatment for 4 days, reaching about 82.7%. This was indicative of a possible G2/M arrest or, alternatively, a failure of cytokinesis that resulted in increase of 4 N or 8 N cell population. And, the second hypothesis was reinforced by the observation that both the increase of DNA endo-reduplication and the accumulation of fibroblasts with DNA content greater than 4 N. This phenomenon of multinucleated cells was also confirmed by nucleo-staining (Fig. 1E) and G-banding (Fig. 3A,B), in which more than 30% of cells failed cytokinesis, whereas almost 100% of fibroblasts failed cytokinesis at 10 μM reversine of 96 h. Chromosome G-banding analysis verified the fibroblasts treated with reversine transformed from diploid/mononuclear (2 N = 54) to tetraploid/binuclear (4 N = 108) and octaploid (8 N = 216) (Fig. 3B). These data suggested that giant multinucleated fibroblasts could exit mitosis without completion of cell division. Thus, polyploidy is a consequence of impaired cytokinesis, which strongly indicates that reversine interferes with cytokinesis and induces polyploidization of fibroblasts.

Reversine has been demonstrated to be an inhibitor of Aurora kinases, and the Aurora kinases regulate mitosis as evidenced by recent work^10^. However, on the contrary, we found that reversine of 5 μM could significantly increase the expression levels of Aurora A and Aurora B in sheep fibroblasts (Fig. 3C). To further confirm the G2/M phase arrest during reversine treatment, four markers of cell cycle progression were detected by Western blotting. Cyclin B1, a G2/M checkpoint regulator involved in promoting mitosis, showed significant higher expression levels in reversine-treated cells (Fig. 2B). Other G2/M regulatory proteins, Cdc2, Cyclin A2 and Cdc25c, also accumulated after reversine treatment. Together, these results clearly suggested that reversine promoted a possible failure in cytokinesis that resulted in the formation of 4 N or 8 N cell population, instead of induction of G2/M phase arrest.

### Rerversine provokes ROS accumulation and mitochondrial dysfunction in fibroblast cells

**Figure 4:**
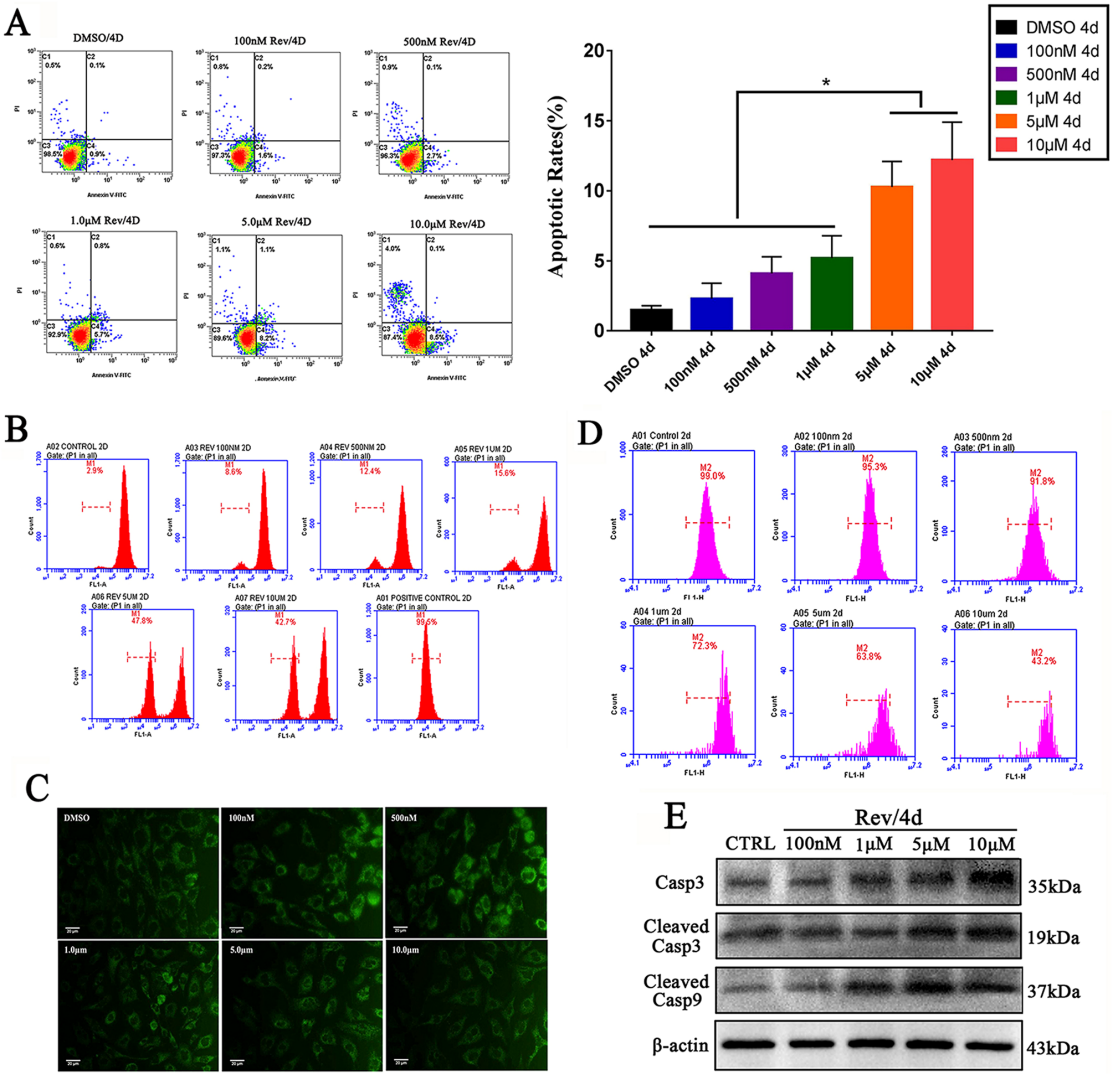
Rerversine provokes ROS accumulation and mitochondrial dysfunction in fibroblasts. (**A**) Cell apoptosis was detected in the reversine treatment groups and the DC group using Annexin V-FITC/PI and flow cytometry. (**B**) Intracellular ROS levels were detected using DCFH-DA by flow cytometry. (**C**) Rh123 fluorescence photomicrographs were observed under an inverted fluorescence microscope. (**D**) MMP was determined using rhodamine 123 by flow cytometry. (**e**) Relative protein expression levels of cell apoptosis related genes were detected by Western blotting.

The percentage of apoptotic and dead cells was significantly increased by high concentration of reversine (Fig. 4A). And, the intracellular ROS levels and the proportion of cells with high ROS were significantly increased after 48 h of treatment with reversine (*P* < 0.01) (Fig. 4B). The fluorescence intensity of Rh123 also diminished in the dose-dependent manner (Fig. 4C). As shown in Fig. 4D, the loss of mitochondrial membrane potential (MMP) was concentration-dependent (*P* < 0.05). Furthermore, as shown in Fig. 4E, the expression levels of the cleavage of caspase 3, caspase 3 and caspase 9 was significantly up-regulated with the increase of reversine concentration. Based on these findings, reversine may promote fibroblasts apoptosis through the mitochondria mediated intrinsic pathway, which has shown similar effect in human colorectal cancer cells^27^.

### Cytokinesis failure triggers Hippo signaling pathway activation and cell autophagy

**Figure 5:**
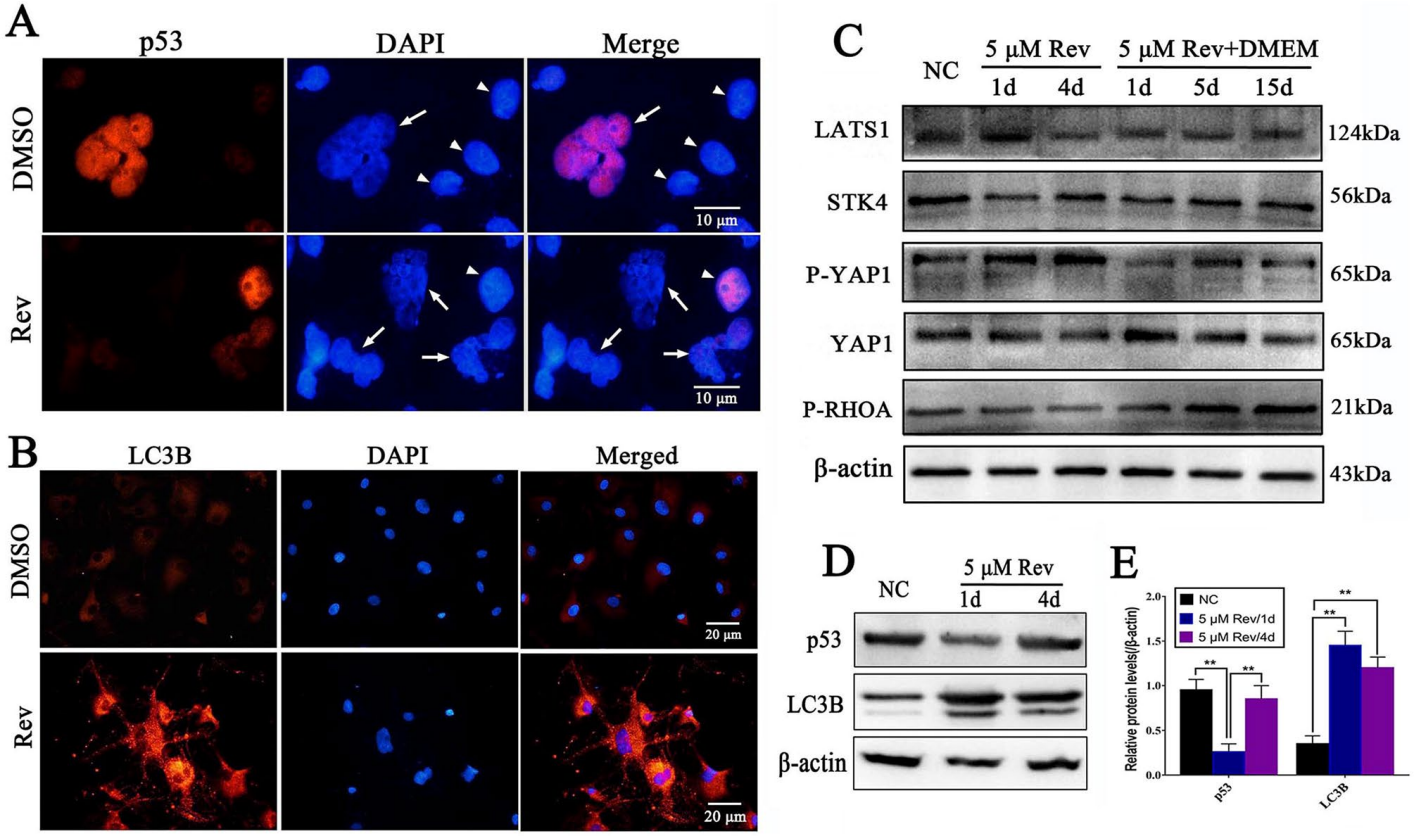
Cytokinesis failure triggers Hippo signaling pathway and P53 inactivation. (**A**) p53 inactivation in reversine treated multinucleated cells, white arrowheads indicate normal fibroblasts, white arrows indicate multinucleated cells. (**B**) Reversine treatment promoted cell autophagy. (**C**) Reversine treatment significantly inhibited RhoA and activated Hippo pathway. (**D**) Western blotting analysis of p53 and LC3B proteins. (**E**) Scanning densitometry was used for the semi-quantitative analysis of western blot analysis for p53 and LC3B.

In this study, the cytoskeleton of tetraploid cells or polyploid cells induced by reversine was completely different from that of diploid cells. Reversine treatment significantly inhibited the level of RhoA (reduced by 50%) and activated Hippo pathway through activation of the kinases LATS1, which phosphorylated and inactivated transcriptional regulators YAP and decreased the level of p53 (Fig. 5A,C,D). Reversine might reduce the contractility of the actin cytoskeleton through inhibiting RhoA and activating Hippo pathway, thus inducing multinucleated cells and cytokinesis failure. What's more, the fibroblasts treated with reversine accumulated small cytoplasmic vacuoles, which was similar to the morphological characteristics of autophagy. As shown in Fig. 5B, a significant increase in the number of red LC-3B puncta after reversine treatment was observed by immunofluorescence analysis. Compared with the control group, reversine treatment resulted in significant increase in LC-3B protein levels (Fig. 5D, E).

### Reversine pretreatment promoted the differentiation potential of sheep fibroblasts

**Figure 6:**
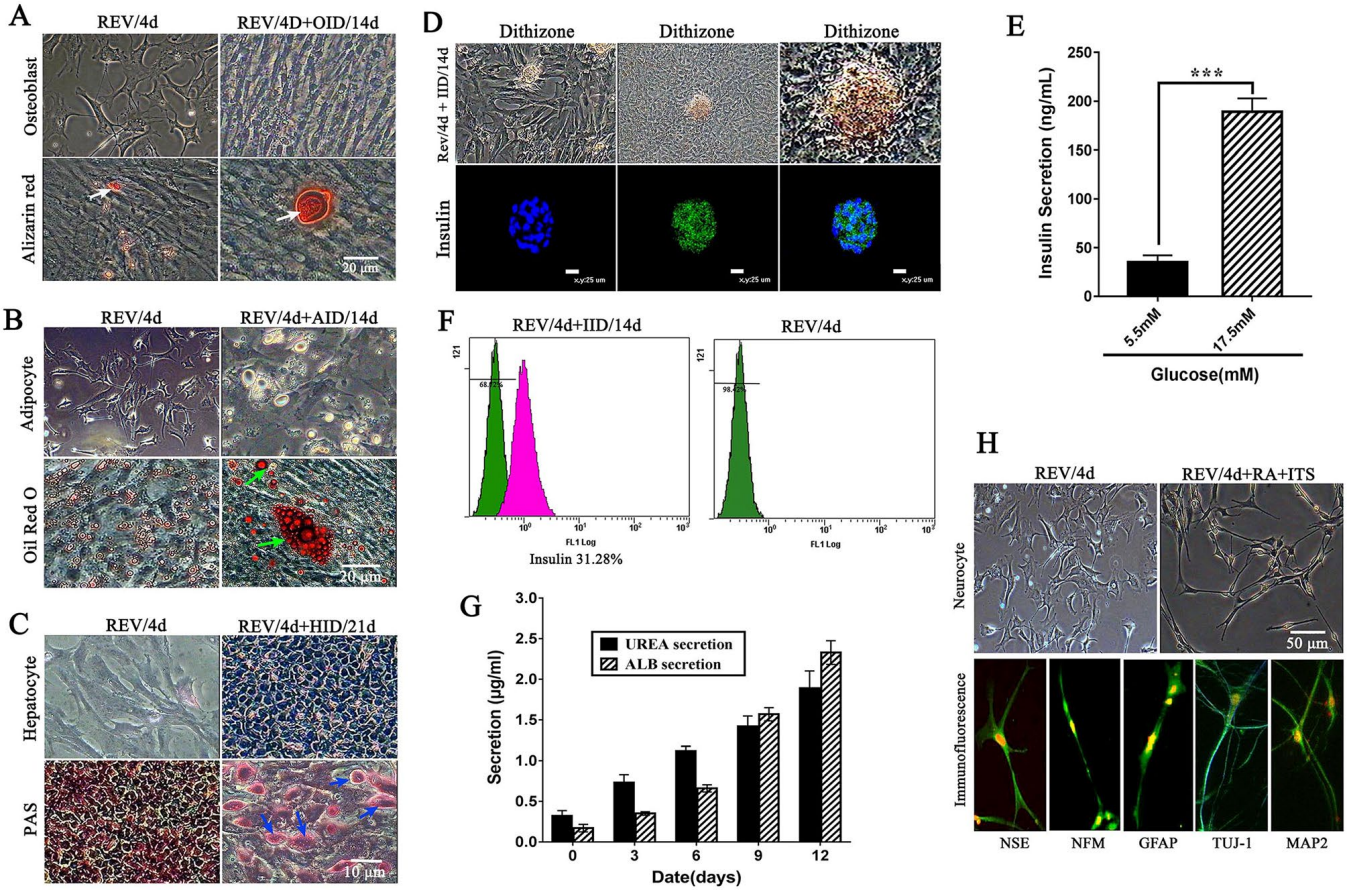
Reversine treatment promoted the differentiation potential of sheep fibroblasts. (**A**) Calcium deposits within induced osteoblasts were positive for Alizarin red staining. (**B**) Intracellular lipid droplets of adipocytes accumulated in cytoplasm and were positive for Oil Red-O staining. (**C**) The hepatogenic differentiated cells were positive by PAS staining assay cultured in HID for 21 days. (**D**) The islet-like clusters were stained scarlet with DTZ fluid and immunofluorescence staining for insulin. (**E**) Glucose induced insulin secretion (Mean ± SEM) in vitro at 5.5 mM and 17.5 mM by ELISA test, ***p < 0.001; (**F**) Only 31.28 ± 2.32% reversine treated fibroblasts were differentiated into insulin-secreting cells. (**G**) Expression of hepatogenic cells marker genes ALB and AFB were analyzed ELISA test. (**H**) Expression of neural specific markers (MAP2, NFM, NSE, GFAP and TUJ-1) were all positive by immunofluorescence staining.

Under different differentiation conditions, fibroblasts treated with reversine could differentiate into three layers cells. The induced osteoblasts were positive for Alizarin Red staining, which confirmed the formation of calcium deposit nodules (Fig. 6A); the induced adipocytes were positive for Oil red O staining (Fig. 6B); In addition, most induced cells exhibited polygonal and mature cuboidal morphology under hepatogenic-inducing (HID) conditions for 21 days, and most of them were positive for glycogen uptake by PAS staining (Fig. 6C); Reversine-induced fibroblasts acquired the differentiation potential for neural lineage, and the expressions of neural specific markers (NSE, NFM, GFAP, TUJ-1 and MAP2) were all positive by immunofluorescence staining (Fig. 6H).

The pancreatic islet-like clusters were stained scarlet with DTZ solution 10 days after induction, and the expression of insulin was detected by immunofluorescence (Fig. 6D). Glucose-stimulated insulin secretion was dose dependent, with a low quantity of insulin secretion (Fig. 6E). Moreover, only 31.28 ± 2.32% of reversine-induced fibroblasts might differentiate into insulin-secreting cells (Fig. 6F). The detoxification of ammonia into urea and the expression of albumin (ALB) protein are the main characteristics for hepatocytes^28^. Compared with the negative control, the levels of urea and ALB in induced fibroblasts were significantly increased in a time-dependent manner (*p* < 0.01, Fig. 6G).

### Reversine promoted the expression of pluripotent and mesenchymal markers

**Figure 7:**
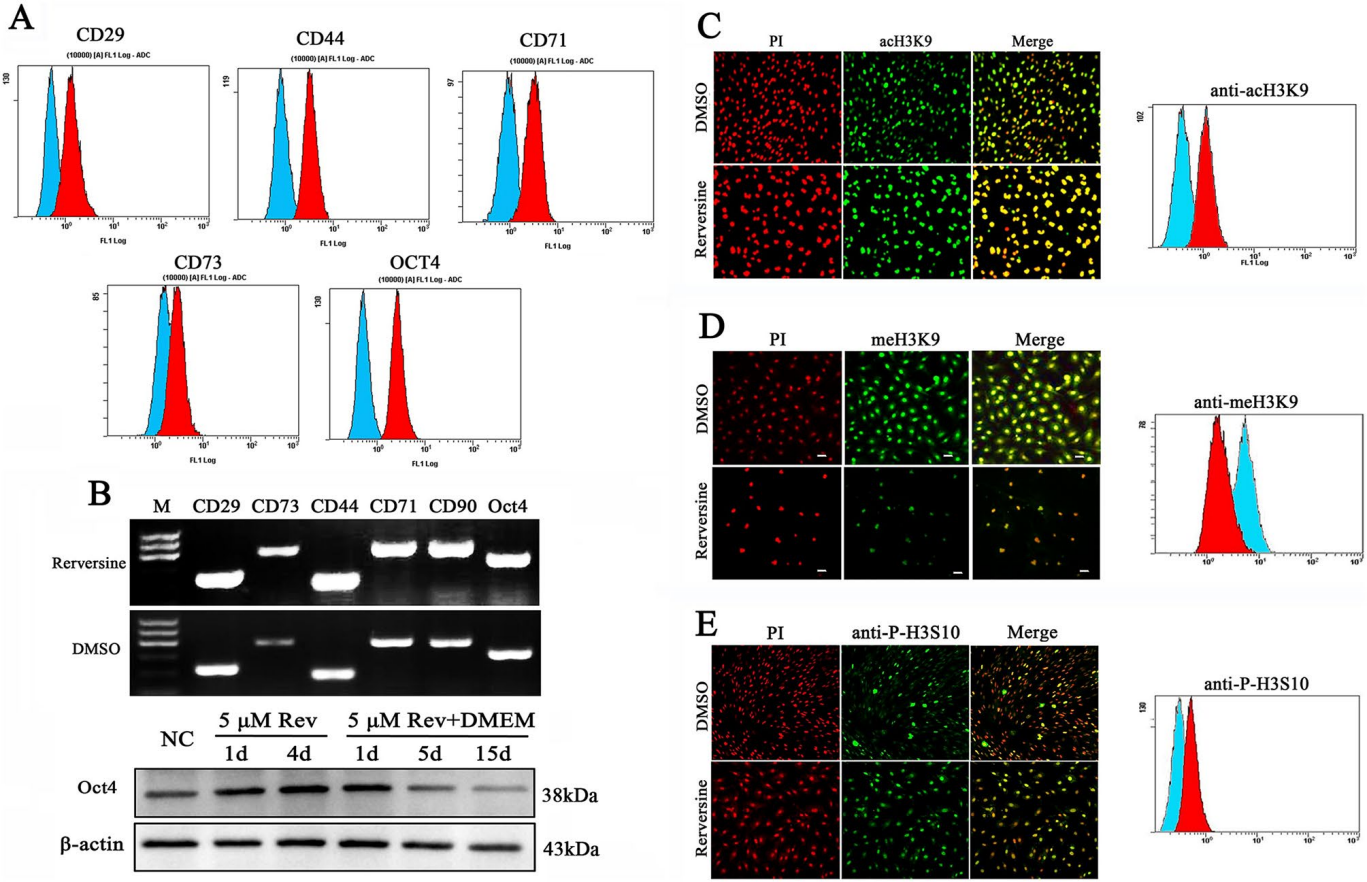
Reversine promoted the expression of pluripotent and mesenchymal markers and epigenetic modification. (**A**) The expression of mesenchymal stem cell markers CD29, CD44, CD71, CD73, and pluripotent marker Oct4 were analyzed by flow cytometry. (**B**) RT-PCR and qRT-PCR were used to examine mRNA expression levels of CD29, CD44, CD71, CD73, CD90 and Oct4. (**C**–**E**) The level of histone acetylation of acH3K9, histone methylatin of meH3K9, and histone phosphorylation of p-H3H10 were analyzed by immunocytochemistry and flow cytometry. PI, Red; anti-acH3K9, anti-meH3K9 and anti-p-H3H10 immunofluorescent, Green.

Reversine significantly promoted the expression of Oct4 (2.5–tenfold) (Fig. 7A,B), but Sox2 and Nanog expression were negative (Fig. S2A,B), which indicated that the activation of Oct4 may play a crucial role in cell multipotency. Importantly, 15 days after reversine removal, reversine-treated fibroblasts gradually reverted to original phenotype, and the expression of Oct4 also returned to a very low level (Fig. 7B). In addition, reversine-treated cells could express specific mesenchymal stem cell surface antigens CD29, CD44, CD71, CD73 and CD90, but hematopoietic cell markers CD34, CD45 and HLA-DR were not detected (Fig. 7A,B). These results demonstrated that reversine could reverse lineage-committed fibroblasts into mesenchymal stem cell-like style.

### Reversine induces epigenetic modification of sheep fibroblasts

Histone modifications such as acetylation, methylation and phosphorylation play very important roles in regulation of gene expression through altering chromatin structure^29^. Reversine treatment caused a significant increase in the level of histone acH3K9 (56.26% ± 4.28 increased, Fig. 7C). In contrast, meH3K9 as the marker of histone methylatin was obviously decreased by 64.25% ± 5.64 (Fig. 7D). After treatment of 5 μM reversine for 24 h, the level of phosphorylation of H3-Ser-10 was remarkably increased (36.34% ± 3.84 increased) (Fig. 7E). The increase of p-H3S10 also reflected the increased activity of Aurora kinases. In summary, the results indicated that the reprogramming of fibroblasts into multipotent progenitor-type cells by reversine might be attributed to the activation of histone acetylation and phosphorylation, and the degression of histone methylation.

### The high-throughput RNA sequencing for mRNA expression discrepancies

**Figure 8:**
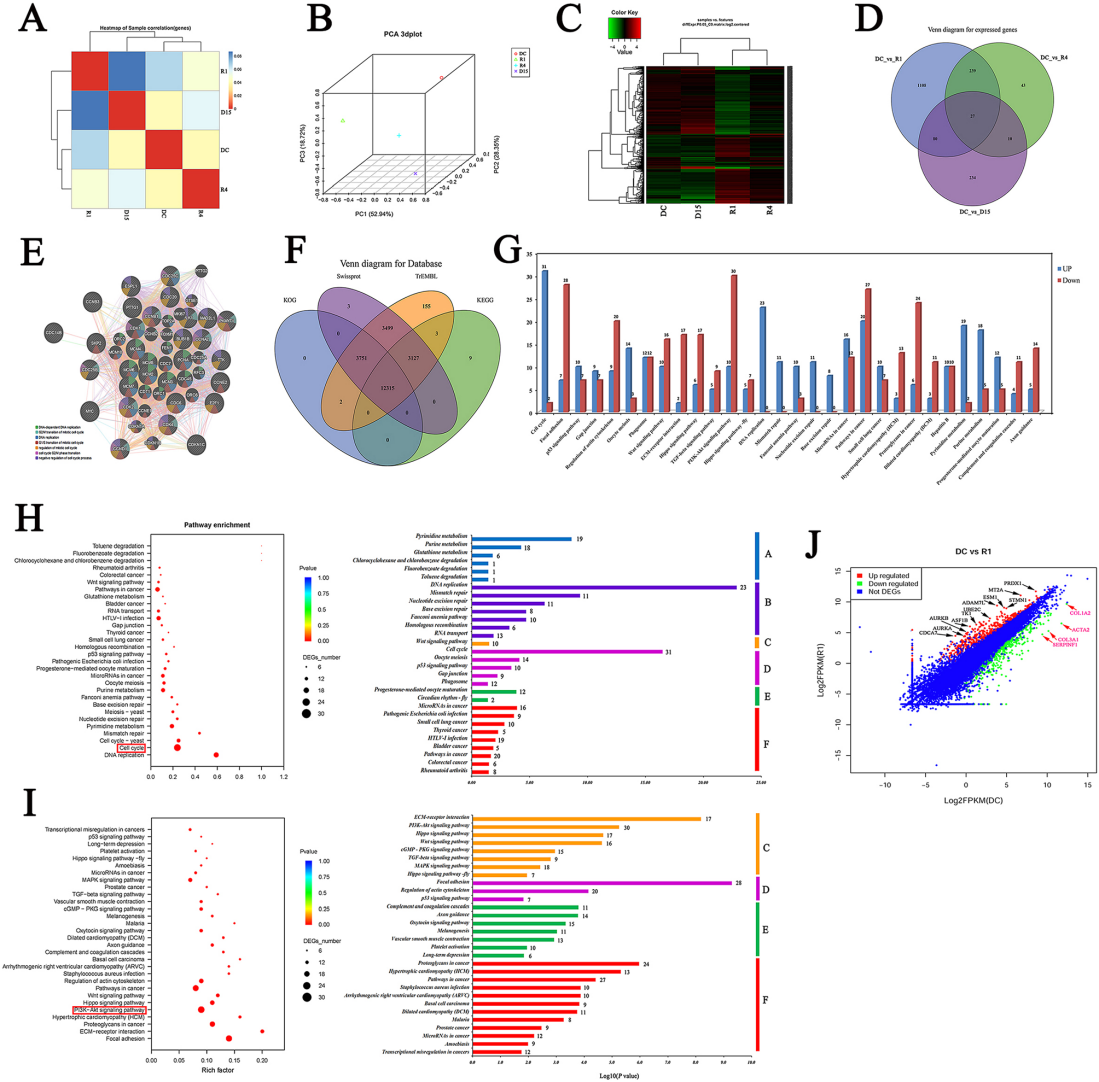
Enriched pathways consisting of DEGs among 4 libraries using RNA-sequencing. (**A**) Heatmap of 4 groups correlation; (**B**) Principal component analysis of 4 samples. (**C**) Heatmap for all DEGs. (**D**) Venn diagram for expressed genes; (**E**) IPA analysis of 27 DEGs found among four groups. (**F**) Venn diagram for 4 databases. (**G**) Top thirty enriched pathways consisting of DEGs between C1 and R1. (**H**) 30 enriched pathways consisting of up-regulated genes were further classified into six categories. (**I**) 30 enriched pathways consisting of down-regulated genes were further classified into four categories. (**J**) Digital expression level of DEGs between C1 and R1. Red points indicate up-regulated DEGs and green points indicate down-regulated DEGs.

Approximate 24.0 Gb of clean reads were acquired from the 4 libraries, and 23,033 unigenes were identified by comparing the genome of *Ovis aries*. By the clustering analysis, R1 and R4 aggregated at one branch, C1 and D15 aggregated at one branch (Fig. 8A). Similarly, R1 possessed the similar expression profile to R4, while C1 was similar with D15 via principal component analysis (PCA) (Fig. 8B) and correlation heat map (Fig. 8C). And, after 15 days of reversine removal, the induced fibroblasts gradually returned to similar phenotype with original control cells. Therefore, it could be shown that sheep fibroblasts were highly sensitive to reversine, which was attributed to its susceptibility and plasticity.

A mass of differentially expressed genes (DEGs) were detected by RNA-Seq, comprising 1,454 genes between R1 and DC, 319 genes between R4 and DC, 351 genes between D15 and DC. The most important 27 DEGs were found among four groups (Fig. 8D). To determine their common molecular and cellular functions, these 27 genes were analyzed by IPA analysis and attributed these functions: DNA Replication, cellular growth and proliferation and cell-to-cell signaling and interaction (Fig. 8E). To demystify the molecular mechanism of fibroblast cells dedifferentiated multipotent mesenchymal progenitor cells induced by reversine, GO and KEGG analyses were performed in the DEGs among R1, R4, D15 and DC (Fig. 8F).

### Pathway enrichment analysis of DEGs among groups

**Figure 9:**
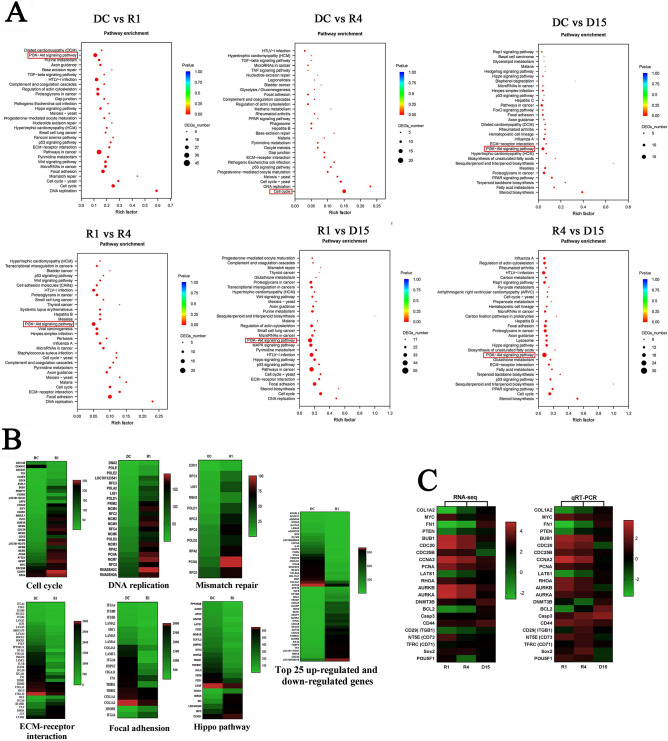
Characteristic comparisons of RNA-Seq dataset among four groups. (**A**) Enriched pathways detection among four group’s DEGs by KEGG analysis. The pathways labeled by red boxes indicate the one enriched the most DEGs compared in two groups. (**B**) DEGs involved in Cell cycle, DNA replication, Mismatch repair, ECM-receptor interaction, Focal adhesion, and Hippo pathway were exhibited, as well as 25 top up-regulated and down-regulated genes between C1 and R1. (**C**) Validation of RNA-Seq data by qRT-PCR. The left profile shows mRNA expression patterns of 22 representative genes detected by RNA-Seq, the right one is the corresponding verification results by qRT-PCR. EF1α was employed as an internal control.

GO and KEGG analysis showed that the six most enriched pathways among four group’s DEGs were associated with PI3K-Akt signaling pathway, Cell cycle, Focal adhesion, HTLV-I infection, Regulation of actin cytoskeleton, and Pathways in cancer. Moreover, up-regulated DEGs were mainly enriched in the PI3K-Akt signaling pathway, while cell cycle was the most enriched pathway for the down-regulated DEGs. Both the PI3K-Akt signaling and cell cycle pathways participate in the regulation of a number of cellular processes, such as regulation of cell proliferation. The pathways labeled with red boxes indicate the most enriched DEGs between the two groups (Fig. 9A). And, 30 enriched pathways consisting of up-regulated genes were further classified into six categories, which were essential for cell characters: Metabolism, Genetic Information Processing, Environmental Information Processing, Cellular Processes, Organismal Systems and Human Diseases (Fig. 8G,H). 30 enriched pathways consisting of down-regulated genes were classified into four categories: Environmental Information Processing, Cellular Processes, Organismal Systems and Human Diseases (Fig. 8G,I). Scatter plots can be useful to identify global changes and trends in gene expression, and the most DEGs between R1 and DC are indicated by arrows (Fig. 8J). Furthermore, the top 25 up-regulated genes and down-regulated genes are depicted as a heat map, respectively (Fig. 9B).

Most DEGs involved in Cell cycle, DNA replication, Pyrimidine metabolism and Purine metabolism were significantly up-regulated in reversine treated fibroblasts (Figs. 8G, 9B). However, these DEGs involved in Mismatch repair, Nucleotide excision repair and Base excision repair were also significantly up-regulated in reversine treated fibroblasts, which means that high concentrations of reversine would cause DNA damage and activated the DNA repair mechanism. In addition, the DEGs involved in Focal adhesion, Regulation of actin cytoskeleton and ECM-receptor interaction were significantly down-regulated in reversine treated fibroblasts (Figs. 8G, 9B). To evaluate the reliability of RNA-Seq data and the rationality of the above explanation, the mRNA expressions of 22 genes were further studied using qRT-PCR. And the results showed that the expression profiles of 22 genes were basically consistent with the RNA-Seq data (Fig. 9C).

## Discussions

Reversine was firstly identified to facilitate dedifferentiation of C2C12 myoblasts into multipotent precursor cells *in vitro*^1^. The present results firstly demonstrated that reversine could reprogram and dedifferentiate sheep fibroblasts into multipotent precursor cells, which could be further induced to differentiate into adipocytes, osteoblasts, β-cells, hepatocytes and neurocytes at high frequency in vitro. Most importantly, the induced β-like cells and hepatocytes had similar metabolic functions as normal cells in vivo. Additionally, reversine could significantly promote the expression of pluripotent gene Oct4 and mesenchymal genes CD29, CD44, CD71, CD73 and CD90, but Sox2, Nanog, CD34 and CD45 were not detected, which authenticated the importance of Oct4 in the acquisition of multipotential mesenchymal stem cell-like cells. Importantly, the expression of Oct4 gradually reduced after reversine removal, supporting the reversibility of reversine-induced change in the sheep fibroblasts.

In general, acetylation of histones H3 and H4 in the regulatory regions of genes is associated with transcriptionally active chromatin, while methylation of histone H3 is correlated with silent inactive chromatin. Recent researches have also highlighted the important role of histone lysine methylations, particularly the methylation of H3K9me2 and H3K27me3 directed transcriptional repression and gene silencing^4^. Reversine significantly increased the acetylation level of H3K9, and decreased the methylation level of H3K9 and H3K27. In addition, reversine treatment significantly induced the phosphorylation of H3-Ser-10, which is considered to be a crucial event for the initiation of mitosis and has been widely used in mammalian cell lines as a mitotic marker. Therefore, reversine induced differentiation of fibroblasts into multipotent progenitor-type cells attributed to the activation of Oct4 and mesenchymal markers, as well as the increase of acetylation and phosphorylation, and decrease of methylation.

Chen et al. reported that reversine treatment induced accumulation of G2/M DNA content resulting in cell cycle arrest before mitosis^1^. In this study, reversine of 5 μM might induce the cell cycle arrest of sheep fibroblasts at G2/M (from 14.4 to 39.1%) and promoted the formation of polyploid cells. However, many fibroblasts treated with reversine cycled rapidly through mitosis, exiting without completing cytokinesis and therefore accumulating as multinucleated cells^30^. Thus, we speculated these cells with 4 N DNA or 8 N DNA content might be multinucleated, instead of just cell cycle arrest in G2 or M phase. To confirm this hypothesis, the multinucleation in fibroblasts treated with reversine were further detected by immunofluorescence and G-band analysis. The results showed that more than 30% of fibroblasts had abnormal cytokinesis after treatment with 5 μM of reversine for 4 days and became multinucleated cells, while almost all cells failed cytokinesis at 10 μM. In addition, we also confirmed four G2/M regulatory proteins, Cyclin B1, Cyclin A2, Cdc2and Cdc25c, all accumulated after reversine treatment.

Aurora B, a part of the chromosomal passenger complex (CPC), involved in the microtubule attachment within the kinetochore of each chromosome by directly phosphorylating multiple proteins within the KNL1/Mis12/Ndc80complexes(KMN)^31^. Aurora A mainly found at duplicated centrosomes, promoted mitotic spindle assembly and cell cycle shift from the S phase to G2 phase^32^. Generally, reversine was considered to be a potent Aurora B kinase inhibitor, which could inhibit the phosphorylation both histone H3 and MPS1 that resulted in chromatin remodeling and perturbation of the chromosome-microtubule attachment^33^. However, the present results showed that reversine of 5 μM significantly increased the expression of Aurora A and Aurora B, promoted nuclear division and the formation of multinucleated cells in sheep fibroblasts. In addition, given that reversine was probably also an inhibitor of dynamin II and non-muscle myosin II, we have to consider the possibility that this inhibitory activity might contribute to the cytokinesis failure.

The conserved Hippo signaling pathway regulates cell proliferation by negatively regulating the transcriptional co-activators YAP/TAZ^34^. Moreover, recent studies have demonstrated that the Hippo pathway is regulated by complex inputs that monitor cell–cell adhesion, cell–matrix adhesion, and contractile tension from the actin cytoskeleton^35, 36^. In this study, reversine treatment significantly inhibited RhoA and activated Hippo pathway through activation of the kinases LATS1, which phosphorylated and inactivated transcriptional regulators YAP and decreased the level of p53. Thus, these results may demonstrate reversine induces multinucleated cells through inhibition of RhoA and activation of Hippo pathway to reduce the assembly and contractility of the actin cytoskeleton, and results in cytokinesis failure. In turn, it also could be that reversine induces cytokinesis failure generating tetraploid and polyploid cells which activates the Hippo pathway. In response to high-dose reversine treatment, fibroblasts undergo successive rounds of aborted mitosis resulting in the accumulation of hyperpolyploid cells, which are prone to apoptosis after catastrophic activation of mitosis.

By KEGG analysis, the six most enriched pathways among four group’s DEGs were PI3K-Akt signaling pathway, Cell cycle, Focal adhesion, HTLV-I infection, Regulation of actin cytoskeleton, and Pathways in cancer. Moreover, most DEGs involved in Cell cycle, DNA replication, Pyrimidine metabolism and Purine metabolism were significantly up-regulated in Reversine treated fibroblasts. These results also suggested that reversine could significantly promote DNA replication and nuclear division of sheep fibroblast. In addition, the DEGs involved in Focal adhesion, Regulation of actin cytoskeleton and ECM-receptor interaction were significantly down-regulated in reversine treated fibroblasts, which might demonstrated why reversine caused cell morphology changes and cell multi-nucleation.

## Conclusion

Reversine could reverse lineage-committed sheep fibroblasts into mesenchymal stem cell-like style and express pluripotent marker gene Oct4 and MSCs-related surface antigens. Moreover, the formation of accumulated cell population with 4 N DNA or 8 N DNA content induced by reversine is attribute to multinucleation of cells rather than just arrested in G2 or M phase. In addition, reversine treatment slightly promoted fibroblasts cell apoptosis via the mitochondria mediated intrinsic pathway.

## Supplementary Information

Supplementary Information.
