# Supplementary material for: Multiple functions of reversine on the biological characteristics of sheep fibroblasts
Source: Sci Rep. 2021 Jun 11;11:12365. doi: 10.1038/s41598-021-91468-w (PMC8196188; doi:10.1038/s41598-021-91468-w)
Supplement: Supplementary file 1 — Supplementary Information. [file 41598_2021_91468_MOESM1_ESM.docx]

**Supplementary Information**

**Title:** Multiple Functions of Reversine on the Biological Characteristics of Sheep Fibroblasts

**Authors:** Yu Guo^1*^, Huan Zhu^1*^, Xiangchen Li^2*^, Cuiyun Ma^1,3^, Tingting Sun^1^, YuanyuanWang^1^, Chunjing Wang^1^, Weijun Guan^3🖂^, Changqing Liu^1,4🖂^

1. School of laboratory medicine, School of Life Sciences, Bengbu Medical College, Bengbu 233000, China;

2. College of Animal science and Technology, Zhejiang A&F University, Hangzhou 311300, China;

3. Institute of Beijing Animal Science and Veterinary, Chinese Academy of Agricultural Sciences, Beijing 100193, China;

4. Department of Neuroscience, University of Connecticut Health Center, Farmington, CT 06030, USA;

***** These authors contributed equally to this work.

**^🖂^**Corresponding. [lcq7813@bbmc.edu.cn](mailto:lcq7813@bbmc.edu.cn) (C.L); [wjguan86@hotmail.com(W.G)](mailto:wjguan86@hotmail.com(W.G))

**Supplementary Figures**

**
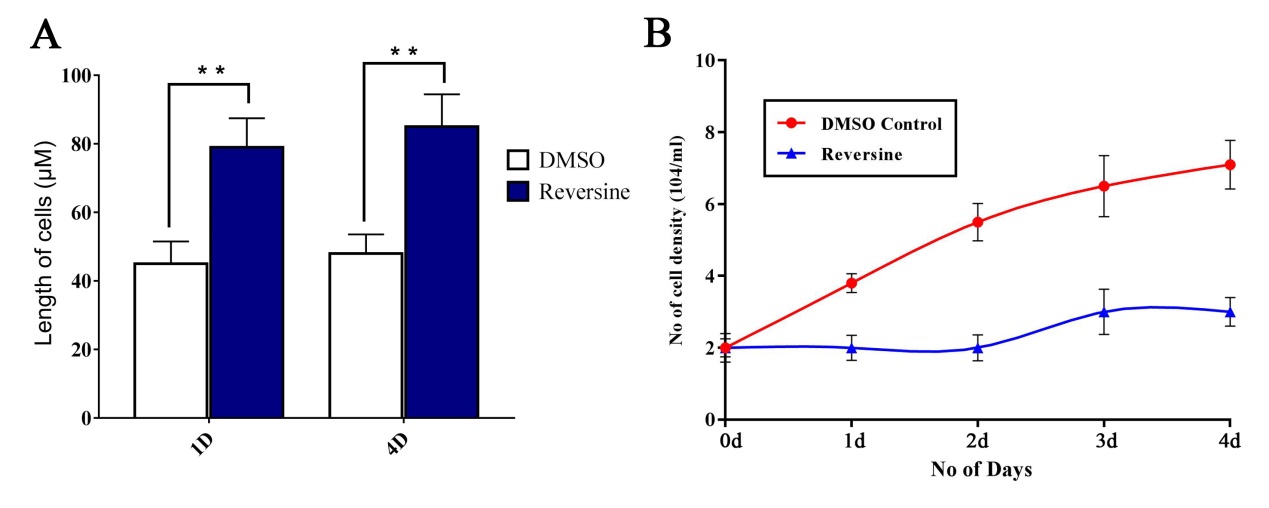
**

**Supplementary Figure S1.** The effects of reversine on the cell length and growth curve. (A) Reversion treatment significantly increased the size and length of cells. (B) Cell viability and cell proliferation were completely inhibited in the presence of reversine in a dose-dependent manner by cell density detection.


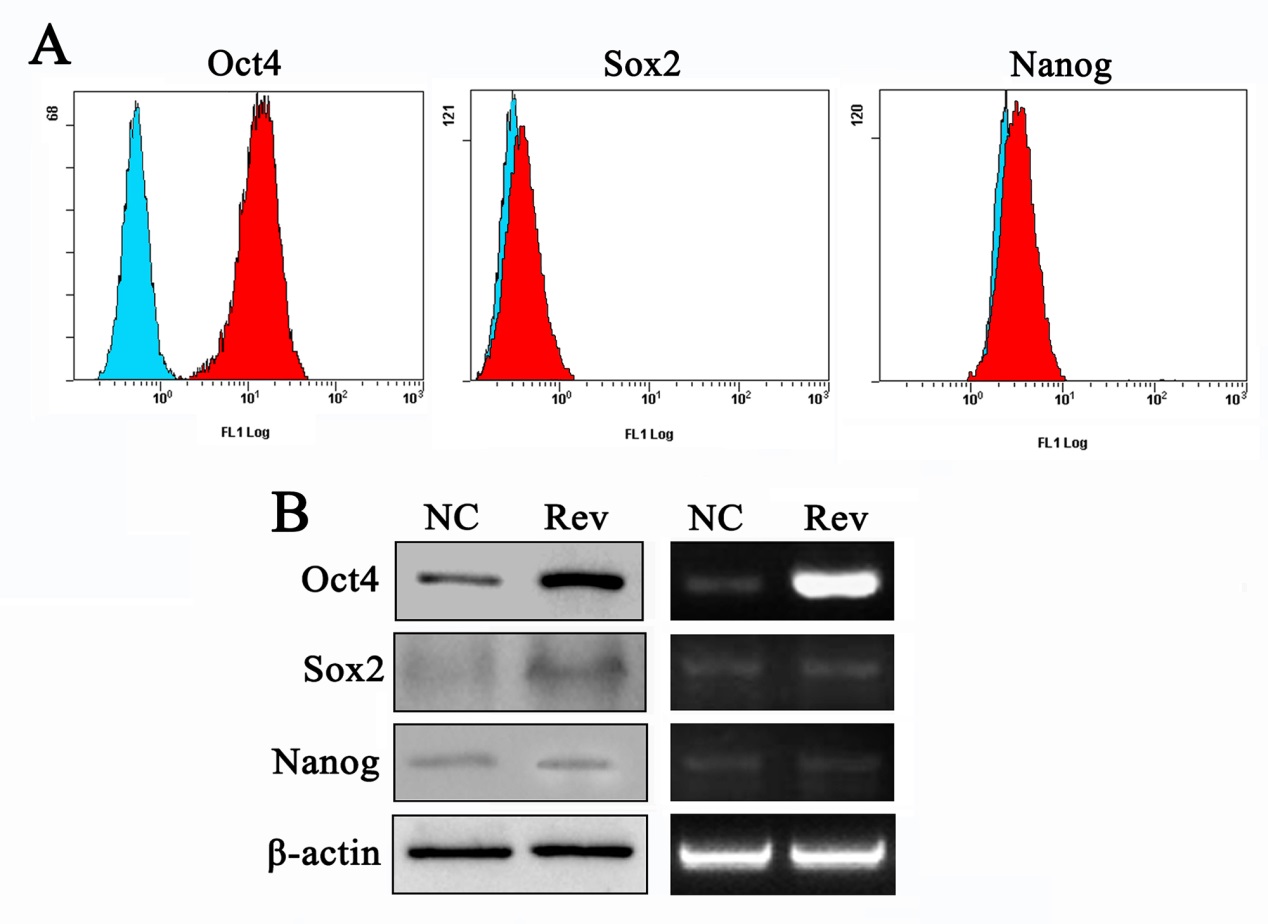


**Supplementary Figure S2.** Expression of Oct4, Nanog and Sox2 in reversine-treated and control cells for the increase of multipotency. (A) Expressions of pluripotent marker Oct4, Nanog and Sox2 was analyzed by flow cytometry. (B) Expression of pluripotent marker genes was analyzed by RT-PCR and Western blotting.

**Supplementary Table S1.** The catalog numbers for all primary antibodies used in this research

| **Antibody Description** | **Manufacturer** | **Catalog numbers** |
| --- | --- | --- |
| anti-NFM | Abcam Biotechnology | ab9034 |
| anti-MAP2 | Abcam Biotechnology | ab11267 |
| Anti-beta III Tubulin(Tuj1) | Abcam Biotechnology | ab78078 |
| anti-NSE | Neuromics | CH23003 |
| anti-GFAP | Neuromics | MO22136 |
| BrdU | Santa Cruz | sc-32323 |
| anti- Aurora B | Abcam Biotechnology | ab2254 |
| anti- Aurora A | Abcam Biotechnology | ab13824 |
| anti-α-tubulin | Abcam Biotechnology | ab18251 |
| anti-CD44 | Abcam Biotechnology | ab19622 |
| anti-CD29 | Abcam Biotechnology | ab5185 |
| anti-CD73 | Santa Cruz | sc-25603 |
| anti-CD71 | Santa Cruz | sc-32272 |
| anti-histone acH3K9 | Santa Cruz | sc-8655 |
| anti-histone meH3K9 | Abcam Biotechnology | ab8898 |
| anti-histone pH3S10 | Santa Cruz | sc-8656-R |
| anti-Oct4(H-134) | Santa Cruz | **sc-9081** |
| anti-Sox2 | Cell Signaling Technology | 3579S |
| anti-Nanog | Cell Signaling Technology | 3580 |
| anti-CCNA2 | Abcam Biotechnology | ab38 |
| anti-CCNB1 | Abcam Biotechnology | ab32053 |
| anti-CDK1 | Bioss | bs-1341R |
| anti-Cdc25c | Bioss | bs-9597R |
| anti-LC3B | Abcam Biotechnology | ab192890 |
| anti-p53 | Bioss | bsm-33058M |
| anti-Collagen I | Bioss | bs-0578R |
| anti-Collagen IV | Bioss | bs-0806R |
| anti-Caspase 3 | Bioss | bs-0081R |
| anti-active Caspase 3 | Bioss | bsm-33199M |
| anti-active Caspase 9 | Bioss | bs-3082R |
| anti-LATS1 | Bioss | bs-2904R |
| anti-STK4 | Bioss | bs-55209R |
| anti-YAP1 | Bioss | bs-3605R |
| anti-Phospho-RhoA | Bioss | bs-5330R |

**Supplementary Table S2.** Primer sequences of pluripotent and mesenchymal specific marker genes

| **Gene** | **Primer Sequences** | **Tm(°C)** | **Length (bp)** |
| --- | --- | --- | --- |
| Oct4 | F:5’-CCGAAAGAGAAAGCGGACGA-3’  R:5’-TGTAGTGAAATGAGGGTCCCG-3’ | 58 | 310 |
| Sox2 | F:5’-CAACGGCAGCTACAGCATGA-3’  R:5’-CCTGGAGTGGGAAGAAGAGGT-3’ | 60 | 286 |
| Nanog | F:5’-GCAGAAATACCTCAGTCTCCAGCA-3’  R:5’-CAGGAGAGTTCACCAAACACCC-3’ | 63 | 221 |
| CD29 | F:5’-TAGAGACTCCAGAGTGCCCC-3’  R:5’-CCGTGTCCCATTTGGCATTC-3’ | 55 | 180 |
| CD73 | F:5’-TTACTGGAGCCCCACACTGA-3’  R:5’-GGAGACACAGGTGCAGGTTT-3’ | 56 | 432 |
| CD44 | F:5’-ACCTCGGATACCAGACACTCA-3’  R:5’-CCGCGAGAATCAAAGCCAAG-3’ | 58 | 136 |
| CD71 | F:5’-GGTGGTTATCGCACCAGAGG-3’  R:5’-TGACAGCGCTGATTTGAGGT-3’ | 56 | 518 |
| CD90 | F:5’-ACGATGCTGATTGTGTGGGA-3’  R:5’-CGGTCACAAGACTTGGCAGA-3’ | 59 | 451 |
